# Interpretations of diffusion-weighted MR imaging by radiology residents in the emergency department: is diagnostic performance influenced by the level of residency training?

**DOI:** 10.1007/s11547-016-0688-4

**Published:** 2016-09-26

**Authors:** Sungjae Lee, Hye Jin Baek, Hyun Kyung Jung, Jin Il Moon, Soo Buem Cho, Bo Hwa Choi, Kyungsoo Bae, Kyung Nyeo Jeon, Dae Seob Choi, Hwa Seon Shin, Dong Wook Kim

**Affiliations:** 1 Department of Radiology, Haeundae Paik Hospital, Inje University College of Medicine, Haeundae-ro 875, Haeundae-gu, Busan, 612-030 Korea; 2 Department of Radiology, Gyeongsang National University School of Medicine and Gyeongsang National University Changwon Hospital, 11 Samjeongja-ro, Seongsan-gu, Changwon, 51472 Korea; 3 Department of Radiology, Gyeongsang National University School of Medicine and Gyeongsang National University Hospital, 79 Gangnam-ro, Jinju, 52727 Korea; 4 Department of Radiology, Busan Paik Hospital, Inje University College of Medicine, 75, Bokji-ro, Busanjin-gu, Busan, 614-735 Korea

**Keywords:** Diffusion-weighted MR imaging, Radiology, Residency training, Diagnostic performance

## Abstract

**Background:**

To evaluate the diagnostic performance of radiology residents’ interpretations for diffusion-weighted MR imaging (DWI) in the emergency department at different levels of residency training.

**Method and materials:**

A total of 160 patients who underwent DWI with acute neurologic symptoms were included in this retrospective study with an institutional review board approval. Four radiology residents with different training years and one attending neuroradiologist independently assessed the DWI results. Discordances between the results of residents and attending neuroradiologist were classified as follows: false positive (FP) and false negative (FN). We also evaluated the diagnostic performance of four residents according to the reference standard.

**Results:**

Overall, the concordance rate was 84.8 %, with a 15.2 % overall discordance rate. There were 83 FN results. The most common misses were small vessel disease (*n* = 55), acute focal infarction (*n* = 10), diffuse axonal injury (*n* = 6), solitary mass (*n* = 5), extraaxial hemorrhages (*n* = 3), posterior reversible encephalopathy syndrome (*n* = 2), and postictal change (*n* = 2). Fourteen FP results were interpreted as hemorrhage and acute infarction. The 4th year resident exhibited the highest diagnostic performance, and the level of training had a significant influence on the rates of concordance (*P* < 0.05). Interobserver reliability was good between the interpretations of the residents and the final interpretations of the attending neuroradiologists.

**Conclusion:**

The level of resident training had a significant effect on their diagnostic performance, and good interobserver reliability was noted between the results of the residents and attending neuroradiologist.

## Background

In the emergency department (ED), patients with acute neurologic deficits are carefully evaluated for a timely diagnosis of intracranial abnormalities by performing neuroimaging studies, such as computed tomography (CT) and magnetic resonance (MR) imaging. Numerous institutions are increasingly performing diffusion-weighted imaging (DWI) for patients with sudden neurologic deficits in the ED to save time and make a correct diagnosis. Furthermore, radiology residents commonly provide preliminary interpretations of neuroimaging studies ordered by the ED at most academic medical centers [1]. Several previous studies reported agreement or discrepancy rates of radiologic examinations [1–5]. However, to date, no objective study has shown a comparison of diagnostic performances with statistical significance for interpreting DWI in radiologic residents with different training years. We hypothesized that the relative inexperience of junior residents may lead to increased discordances of their interpretations and that the level of residency training may be related to the discordance rate. Therefore, the purpose of this study was to retrospectively assess the rates of diagnostic discordances for DWI in ED between the interpretations of radiology residents and the final interpretations of an attending neuroradiologist. We also sought to evaluate the diagnostic performance of radiology residents at different levels of residency training.

## Materials and methods

### Study population

A review of the database of our institution identified 297 consecutive patients who underwent DWI in the emergency department between September 2015 and December 2015. We then selected 213 of these 297 patients with acute neurologic symptoms using electronic medical charts and picture archiving and communicating system (PACS). Of these 213 patients, 53 were excluded due to inadequate medical records (*n* = 24); poor image quality, including motion artifacts or susceptibility artifacts (*n* = 19); and inadequate diagnosis by only DWI (*n* = 10). The final 160 patients who were included in this study comprised 84 males and 76 females (age range, 28–86 years; mean age, 63.4 years). Retrospective data collection and analysis were performed according to our local institutional review board (IRB) guidelines after its approval, and the IRB determined that patient approval and informed consent were not required for reviewing images and records.

### Imaging acquisition

MR imaging was performed using a 3-T system (Achieva; Philips Medical Systems, Best, The Netherlands) with a 32-channel head coil. Our DWI protocol included the following sequences: axial DWI, axial fluid-attenuated inversion recovery (FLAIR), and axial T2*-weighted gradient echo image (GRE). The parameters for echo-planar DWI were as follows: b values, 0 and 1000 s/mm^2^; repetition time (TR)/echo time (TE) msec, 6000/83; field of view (FOV), 21 cm; section thickness, 5 mm; matrix, 128 × 128; number of slices, 24; and acquisition time, 2 min 03 s. The parameters for FLAIR were as follows: TR/TE msec, 10,000/120; FOV, 21 cm; section thickness, 5 mm; matrix, 256 × 152; number of slices, 24; and acquisition time, 2 min 30 s. The parameters for T2*-weighted GRE were as follows: TR/TE msec, 529/16; FOV, 21 cm; section thickness, 5 mm; matrix, 324 × 193; number of slices, 24; and acquisition time, 1 min 43 s.

### Imaging analyses and reference standard

In our institution, we had a total eight radiologic residents in the radiologic department. Of these eight residents, four residents could not join in this study, because of a secondment for outreach education of interventional radiology, personal reason, and training schedule. Finally, four radiology residents at different training year levels interpreted the DWI of all patients. At the time of this study, the 1st year resident completed one-half of her 1st year of training, had 2 months of neuroradiology experience interpreting both CT and MR imaging, and participated in neuroradiology teaching conferences, including staff lectures and interesting case presentations. All residents evaluated images and recorded the following information: the presence of abnormalities, location of detected abnormalities, and presumed diagnosis. An attending neuroradiologist (H.J.B. with 6 years of experience in brain, head, and neck imaging) also interpreted the same images independently, and her interpretations were used as the reference standard. All reviewers were blinded to patient clinical data, except the reason for the examination. Discordance between the resident’s and staff’s interpretations was classified as either false positive (FP; e.g., misinterpreting normal images as abnormal) or false negative (FN; e.g., failure to diagnose an abnormality). In addition, concordance was classified as true positive (e.g., agreement of results between resident and staff) or true negative (e.g., negative finding).

### Statistical analysis

Data were analyzed using Fisher’s exact test for evaluating discordance rates. The diagnostic indices (sensitivity, specificity, positive and negative predictive values, and accuracy) of each resident were also calculated. A receiver-operating characteristic (ROC) curve was constructed to evaluate the diagnostic performance of each resident with the largest Az value. Interobserver agreement between residents and attending neuroradiologist was assessed by kappa (κ) statistics. The κ statistics results were interpreted as follows: κ values ranging from 0.21 to 0.40 indicated fair agreement; 0.41 to 0.60 indicated moderate agreement; 0.61 to 0.80 indicated good agreement; and 0.80 to 1.00 indicated very good agreement. All statistical analyses were performed with statistical software (SPSS, version 19.0, SPSS, Chicago, IL, USA; MedCalc, version 9.0, MedCalc Software, Mariakerke, Belgium), and *P* values less than 0.05 were considered statistically significant.

## Results

Of the 160 patients, various neurologic symptoms were noted, including headache (*n* = 45, 28.1 %), dizziness or vertigo (*n* = 38, 23.8 %), motor weakness (*n* = 36, 22.5 %), sensory change (*n* = 28, 17.5 %), and cranial nerve symptom (*n* = 13, 8.1 %).

Of the 160 DWI scans, 96 (60 %) were abnormal and 64 (40 %) were considered normal. The locations of abnormalities were as follows: cerebral hemisphere, including the cortex and white matter (41/96, 42.7 %); deep gray matter (18/96, 18.7 %); brainstem (16/96, 16.7 %); extraaxial spaces (14/96, 14.6 %); and cerebellum (7/96, 7.3 %). Table 1 demonstrates the range of radiologic diagnoses that were made given the interpretation by the attending neuroradiologist, as a reference standard. The most common diagnosis was acute infarction (42/96, 43.8 %) followed by small vessel disease (27/96, 28.1 %).

**Table 1:** Radiologic diagnosis made at diffusion-weighted MR imaging in the emergency department

Final diagnostic interpretation	Total no. of cases (*n* = 96)
Acute infarction	42 (43.8)
Small vessel disease (white matter hyperintensities, microbleeds, old lacunar infarcts)	27 (28.1)
Intraparenchymal hemorrhage	8 (8.3)
Subdural hemorrhage	5 (5.2)
Subarachnoid hemorrhage	4 (4.2)
Intraventricular hemorrhage	3 (3.1)
Diffuse axonal injury	3 (3.1)
Solitary mass	2 (2.1)
Posterior reversible encephalopathy syndrome	1 (1)
Postictal change	1 (1)

Data presented in parentheses are percentage of each item

Overall, the concordance rate was 84.8 %, with a 15.2 % overall discrepancy rate. Fortunately, most discrepancies were insignificant. In total, 83 FN results were noted. The missed diagnoses of residents were small vessel disease (*n* = 55, 66.3 %), acute focal infarction (*n* = 10, 12 %), diffuse axonal injury (*n* = 6, 7.3 %), solitary mass (*n* = 5, 6 %), extraaxial hemorrhages (*n* = 3, 3.6 %), posterior reversible encephalopathy syndrome (*n* = 2, 2.4 %), and postictal change (*n* = 2, 2.4 %). Fourteen FP results were interpreted as hemorrhage and acute infarction. Table 2 summarizes the total number of concordances and discordances, regarding each level of radiologic residency training. The rate of discordance was the highest for the 1st year resident (17.6 %), and the level of training had a significant influence on the diagnostic accuracy (*P* < 0.05) (Table 3).

**Table 2:** Concordances and discordances of diffusion-weighted MR studies by level of training

Level of training	Correct diagnosis		FP results	FN results	Total no. of discrepancies
	TP	TN			
R1	74 (46.2)	58 (36.2)	6 (3.8)	22 (13.8)	28 (17.6)
R2	72 (45)	61 (38.1)	3 (1.9)	24 (15)	27 (16.9)
R3	71 (44)	63 (39)	1 (0.6)	25 (15.6)	26 (16.2)
R4	77 (48)	72 (45)	4 (2.5)	12 (7.5)	16 (10)

Data are number of examinations; numbers in parentheses are percentages

*FN* false negative; *FP* false positive; *NPV* negative predictive value; *PPV* positive predictive value; *TN* true negative; *TP* true positive

**Table 3:** Diagnostic performance of radiology residents interpretations for diffusion-weighted MR images

Year of training	A_z_ value	Sensitivity (%)	Specificity (%)	PPV (%)	NPV (%)	Accuracy (%)
R1	0.839 (0.770, 0.892)	77.1	90.6	92.5	72.5	82.5
R2	0.852 (0.787, 0.903)	75	95.3	96	71.8	83.1
R3	0.862 (0.799, 0.911)	74	98.4	98.6	71.6	83.8
R4	0.906 (0.850, 0.947)	87.5	93.8	95.5	83.3	90

A_z_ indicates the largest area under the ROC curve

Numbers in parentheses are 95 % confidence intervals

*NPV* negative predictive value; *PPV* positive predictive value

**Fig. 1:**
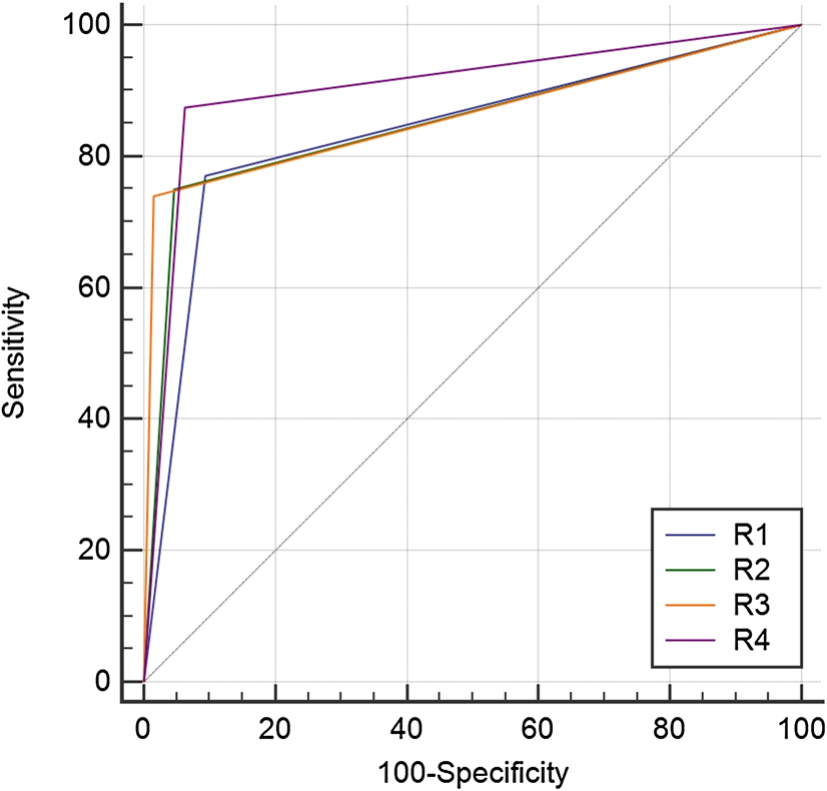
Diagnostic performance of four radiology residents’ interpretations for diffusion-weighted MR images. Diagonal line = 50 % of the area under the ROC curve and also refers to a hypothetical marker that has no discriminatory power for diagnosing diffusion abnormalities

Among residents with different years of training, the 4th year resident exhibited the highest diagnostic performance with the largest area under the ROC curve (0.906; 95 % confidence interval: 0.850, 0.947), a sensitivity of 87.5 %, and a specificity of 93.8 %. The diagnostic performance of each resident was demonstrated by comparison of ROC curves in Fig. 1. A good degree of interobserver reliability was noted between all residents and attending neuroradiologist (*P* < 0.0001 and Table 4).

**Table 4:** Assessment of interobserver reliability for interpreting the diffusion-weighted MR images between radiology residents and attending neuroradiologist

Year of training	Agreement	κ value	*P* value
R1	132/160	0.650	<0.0001
R2	133/160	0.667	<0.0001
R3	134/160	0.681	<0.0001
R4	149/160	0.796	<0.0001

## Discussion

In ED, patients with acute neurologic deficits are carefully evaluated for the early diagnosis of intracranial abnormalities with neuroradiologic examinations, including CT and MR imaging. However, routine MR imaging can be a time-consuming assessment for these patients. The previous studies demonstrated that DWI is an effective imaging modality that has reliable sensitivity and specificity in patients with acute neurologic problems [5, 6]. Therefore, many institutions have used DWI in combination with FLAIR and T2*-weighted GRE as a timesaving substitute for routine brain MR imaging to make a timely diagnosis. At most academic medical centers, physicians in the ED request urgent DWI studies for patients with acute neurologic deficits and radiology residents are often responsible for providing preliminary interpretations of those studies before the final interpretations of the neuroradiologist become available [1]. However, the physicians’ need for rapid and accurate diagnoses of neuroimaging studies can conflict with the need for radiologic residents to acquire clinical experience and confidence [3]. To maintain proper resident training, meticulous analyses of residents’ misinterpretations and discordances between residents’ and final interpretations are mandatory, because residents’ interpretations may impact patient management and treatment planning in the ED.

In this study, we retrospectively assessed the rates of diagnostic concordances and discordances for DWI in ED between the interpretations of radiology residents and the final interpretations of attending neuroradiologist. We also evaluated the diagnostic performance of radiology residents at different levels of residency training.

In this study, the overall rate of concordance was 84.8 % with 15.2 % of overall discordance rate, and most of discordances were insignificant. Although the discordance rate between the initial interpretations of head CT scans by ED physicians and the final interpretations by radiologists has been found to be nearly 39 % [7], the discordance rate of residents is much lower in this study. This discordance rate is higher than that reported by investigators who examined radiology residents’ interpretations of head CT scan or brain MR imaging studies below 5 % [1, 2, 8–13]. The discordance rate of our study is better than that previously reported for imaging modalities of other body sections, where disagreement rates as high as 26 % were reported for chest radiography [14]. These differences may be produced by the relatively small number of enrolled patients and participating radiology residents as well as the selected imaging modality for the study. Our institution is a medium-sized academic medical center with 2 radiology residents at each level of training. Of a total of 8 residents, only 4 residents participated in this study due to their training schedule.

Consistent with the previous results [1, 3], we found that the discordance rate for the 1st year resident was significantly greater than those of 2nd, 3rd, and 4th year residents. Similarly, the diagnostic performance of high-level residents was also significantly greater for DWI interpretations. Our results suggest that clinical and educational experiences may play a role in interpreting imaging studies. Although individual differences exist, confident interpretation and decision-making is one of the most important educational and clinical experiences for radiology residents [1, 14].

**Fig. 2:**
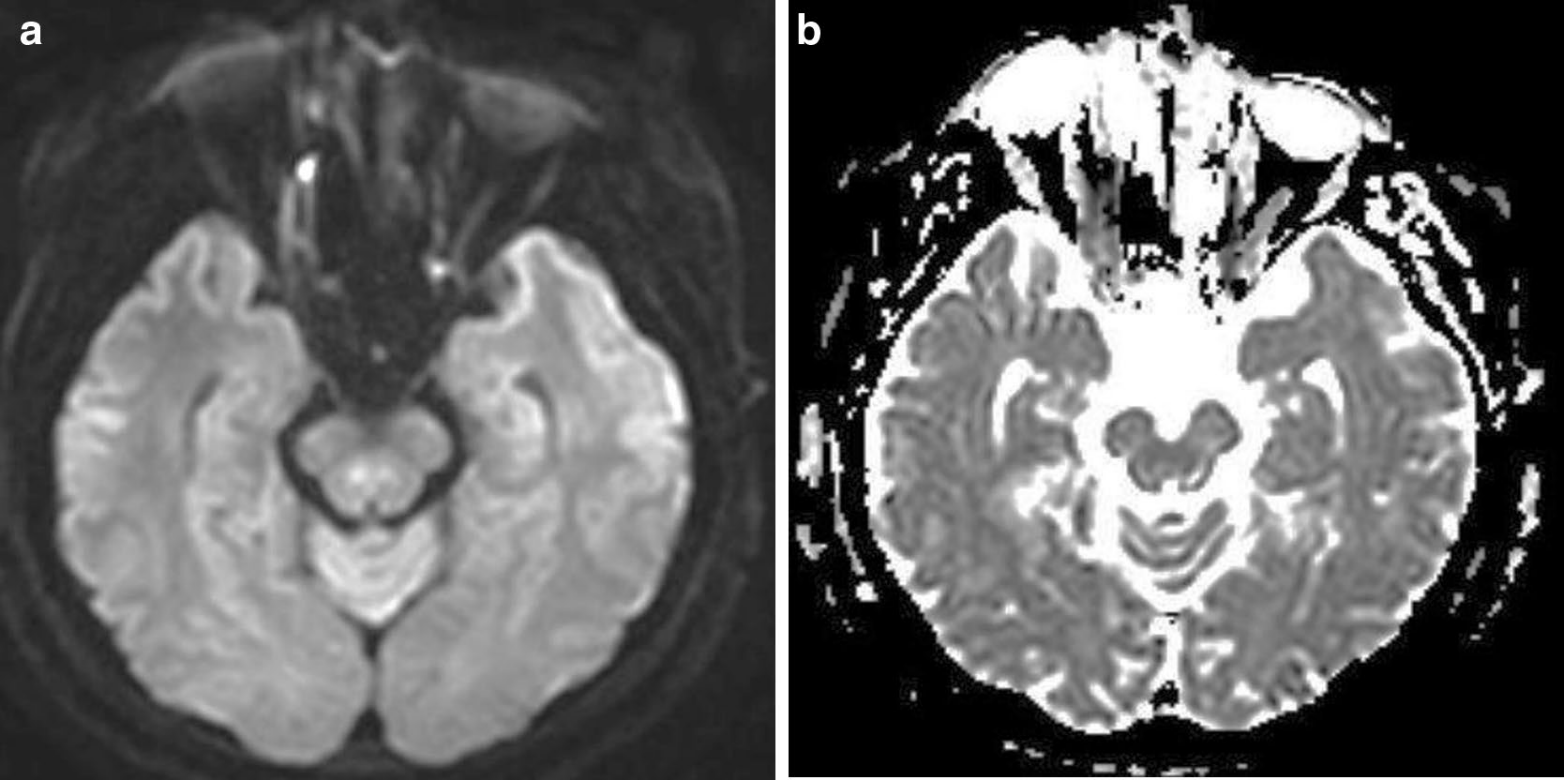
False-positive case. A 52-year-old male patient with sudden onset diplopia. **a, b** Small region of hyperintensity with equivocal ADC change is noted in the right median portion of midbrain. This finding represents a physiologic hyperintensity by anisotropy of the superior cerebellar peduncle

**Fig. 3:**
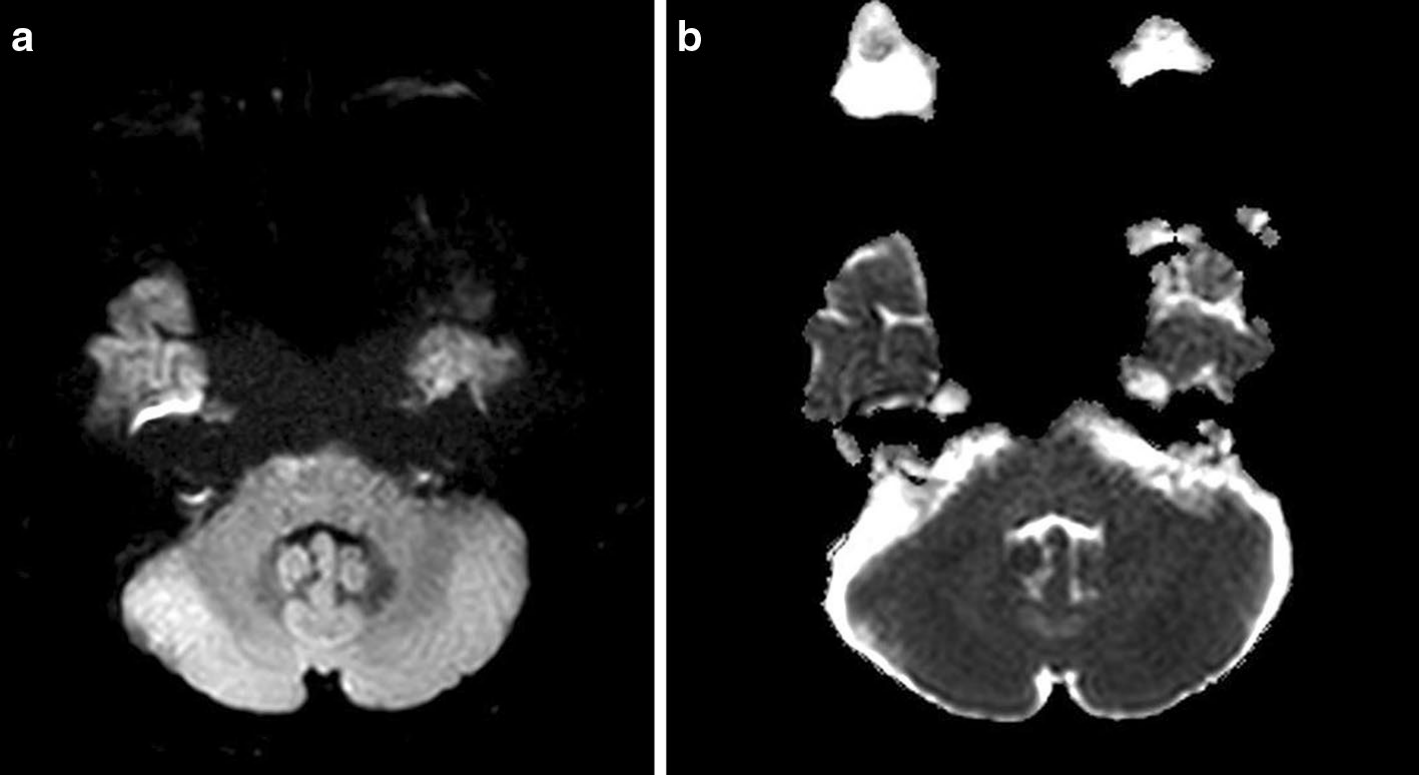
False-positive case. A 41-year-old female patient with vertigo. **a, b** A small region of hyperintensity without ADC change is seen in the anterior portion of the right mid pons. This is a pseudolesion by suspected susceptibility artifact

Of 160 cases, 14 FP findings with misinterpretations of acute focal infarction and focal hemorrhage were noted. All of the 14 FP lesions were small in size, and these errors were related to artifacts intrinsic to DWI, such as physiologic hyperintensity by anisotropy or T2 shine-through effect (Figs. 2, 3). Interpretations can be made easier if radiology residents keep these errors in mind.

**Fig. 4:**
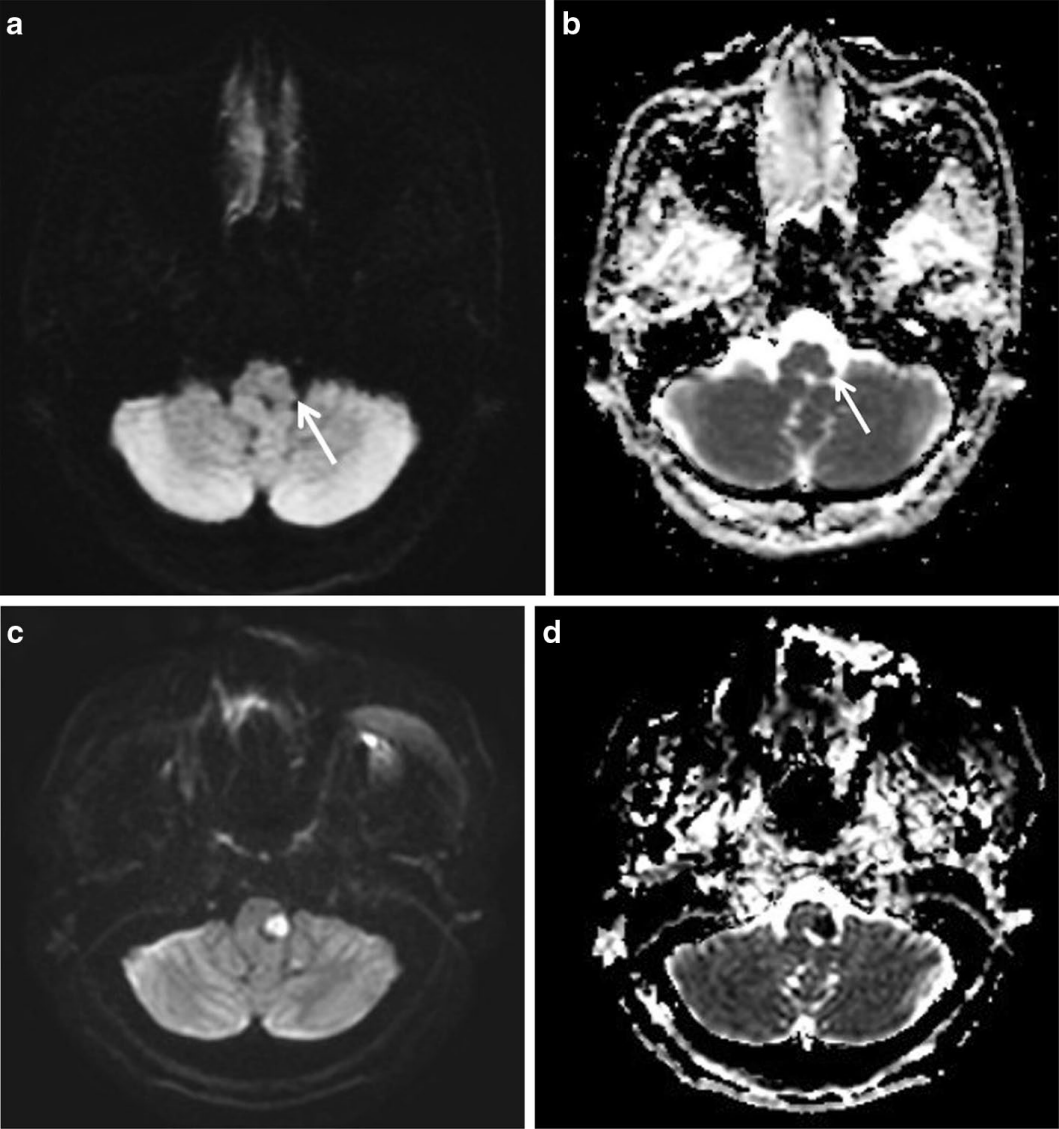
False-negative case. A 47-year-old female patient with acute facial numbness. **a, b** A tiny diffusion restriction is noted in the lateral portion of the left sided medulla (*arrows*). This finding is characteristic of acute lateral medullary infarction. However, all residents missed this lesion and interpreted the image as normal. **c, d** Two days after admission, the lesion increases in size with more conspicuous ADC change

**Fig. 5:**
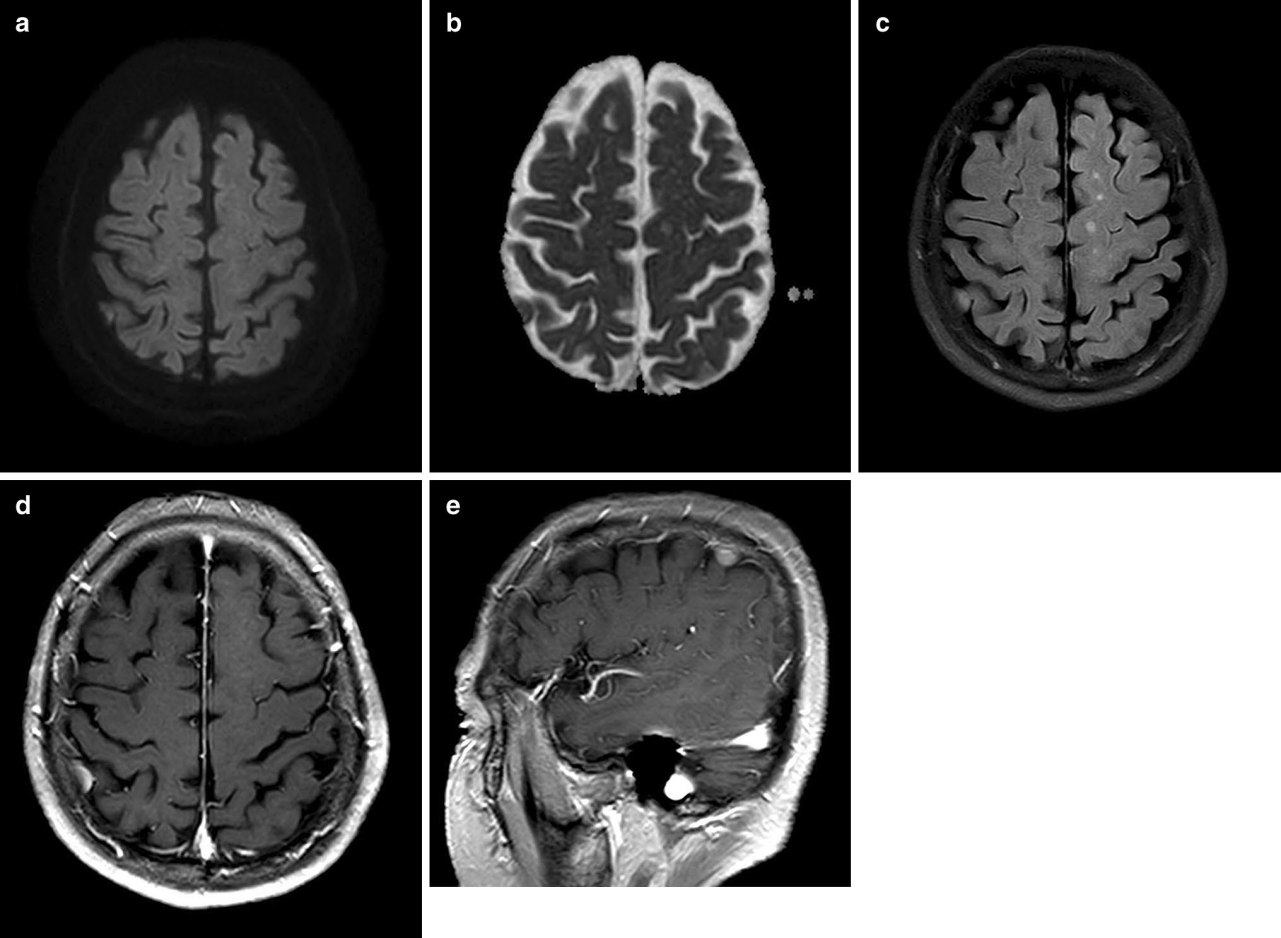
False-negative case. A 68-year-male patient with sudden onset vertigo. **a, b** Small extraaxial mass is located in the right high parietal convexity without diffusion restriction. **c** The lesion exhibits isointensity on FLAIR image. However, all residents interpret DWI as normal. **d, e** On contrast-enhanced axial (**d**) and sagittal (**e**) T1-weighted images from the following day, the lesion exhibits homogenous enhancement, suggesting convexity meningioma

Among FN cases, grade I small vessel disease was the most common missed diagnosis followed by acute focal infarctions (Figs. 4, 5). Fortunately, these FN cases were not significant. All of these lesions were relatively small in size and number; thus, a more careful imaging evaluation may improve the diagnostic accuracy of resident’s interpretations. In the case of acute focal infarction, the meticulous evaluation of the apparent diffusion coefficient map can be helpful to make an accurate diagnosis.

In addition, our study showed good interobserver reliability between the interpretations of residents and attending neuroradiologist. This result suggests the possibility that the resident’s interpretations of DWI may be reliable in the patient with acute neurologic deficits who visit the ED before the final interpretations of the subspecialized neuroradiologist become available. Interestingly, κ values for interobserver reliability between residents and attending neuroradiologist tended to increase as the level of the compared resident increased. These results may indicate that clinical experiences during residency training can be an important factor for imaging interpretation.

There are several limitations of this study. First, a relatively small number of enrolled patients and participating residents at each level of residency training were included in this study. Therefore, our study had a weakness for generalization. Second, we did not investigate the clinical outcome during patient’s total hospital stay, because this study was retrospective. Thus, we could not analyze the final effect of residents’ interpretations on patients’ clinical outcomes. Third, insufficient evaluations were available for infratentorial lesions, because DWI examinations performed at our institution did not focus on the posterior fossa with thin-section slices. Finally, we used the final interpretation of only one attending neuroradiologist as the reference standard. Ultimately, we could not evaluate the possibility of FP and FN results made by the attending radiologist. However, this method was used successfully in the previous studies [1, 3, 8]. To valid our result, further studies with additional attending neuroradiologists or more experienced senior attending neuroradiologists are required.

## Conclusion

In conclusion, high-level residents exhibited a better diagnostic accuracy for interpreting DWI ordered from the ED compared with junior residents, and the level of resident training had a significant effect on their diagnostic performances. Good interobserver reliability was noted between the interpretations of residents and attending neuroradiologist. Therefore, radiology residents can safely provide interpretations of DWI requested by the ED, and efforts to focus on detecting small lesions can be helpful to reduce residents’ errors.
